# The effect of a shoulder injury prevention programme on proprioception and dynamic stability of young volleyball players; a randomized controlled trial

**DOI:** 10.1186/s13102-021-00300-5

**Published:** 2021-06-30

**Authors:** Mostafa Zarei, Saeed Eshghi, Mahdi Hosseinzadeh

**Affiliations:** 1 Sport Rehabilitation and Health Department, Faculty of Sports Sciences and Health, Shahid Beheshti University, Velenjak Square, Evin, 1983963113 Tehran, Iran; 2 Department of Sport Injuries and Corrective Exercises, Sport Sciences Research Institute, Tehran, Iran

**Keywords:** Proprioception, 11 + Shoulder, Injury Prevention, Volleyball

## Abstract

**Background:**

The “FIFA 11 + Shoulder” programme has been reported to reduce the incidence of upper extremity injuries among soccer goalkeepers. It has also been recommended for overhead sports. The purpose of this study was therefore to investigate the effect of an 8-week “FIFA 11 + shoulder” (11 + S) programme on shoulder joint position sense (JPS), threshold to detect passive motion (TTDPM) and upper quarter Y Balance Test in young male volleyball players.

**Methods:**

Thirty-two healthy young elite male volleyball players (17.49 ± 1.47 years) participated in this quasi-experimental study. Participants, recruited from two clubs participating in Iranian premier league, were randomly assigned into two groups; (1) the intervention group who performed the “FIFA 11 + shoulder” programme as their warm up protocol, three times per week, and (2) the control group who kept their routine warm up protocol meanwhile. Proprioception tests including JPS and TTDPM of internal and external rotator muscles of the dominant shoulder were recorded via the isokinetic system pro 4. The upper quarter Y Balance Test determined the shoulder dynamic stability.

**Results:**

No statistically significant differences were observed for JPS and TTDPM of shoulder internal and external rotator muscles; shoulder stability however significantly increased only in the intervention group (*p* = 0.03, ηp^2^=0.02).

**Conclusion:**

Upper quarter dynamic stability improvement due to the 11+S programme leads to volleyball players’ performance and may therefore contribute to a reduction in risk of sustaining injury if applied long-term.

**Trial registration:**

The trial was retrospectively registered atIranian Registry of Clinical Trials with the number of IRCT20201030049193N1 at 04/12/2020.

## Background

Repetitive high-demand throwing activities such as spikes and services put extra pressure on the shoulder joint of the volleyball players. The ability to perform these movements smoothly requires high coordination in the muscles around the shoulder. This harmony is achieved through the proprioception [1–3]. Proprioception is a comprehensive word for the sense of motion that receives sensory input from the spindle, tendon and joint receptors, which includes our ability to locate our organs in space (joint position sense) and our ability to detect motion (kinesthetic) and determines the direction, intensity, and velocity of joint movement [4]. Proprioception plays an important role in providing dynamic stability of the shoulder joint as well as muscle coordination in overhead sports such as volleyball [5]. Dynamic stability is the ability of an athlete to stabilize the center of mass of the body while rotating the distal extremity. Greater dynamic stability of the joint requires appropriate force applied through the muscle tension. The size of these forces must be properly coordinated.

Proprioception deficiency can impair muscle nerve control, which can lead to muscle imbalance and joint instability [6]. Contemori et al. [7] stated that proprioception defects can alter the function of the dominant arm of volleyball players exposing them to acute or chronic injury. Moreover, Allegrucci et al. [8] stated that defect in dominant shoulders kinesthesia of thrower players is a mechanism for sustaining shoulder instability. Identifying effective interventions to enhance proprioception is therefore, important for the prevention of injuries and the recovery of function in athletic rehabilitation and musculoskeletal physiotherapy [9].

Isokinetic dynamometry is suggested as one of the ways to evaluate the proprioception. The isokinetic dynamometer is one of the most reliable tools for measuring shoulder proprioception through both the active and passive protocols [10]. Many researchers have used this device to assess proprioception. Lee et al. [11] and Sales et al. [12] evaluated the proprioception of shoulder internal and external rotator cuff muscles using the isokinetic dynamometer.

Although the effect of exercise training on proprioception is not clear, exercise can enhance proprioception by modifying the sensitivity of muscle spindles as well as increasing subject attention to the joint position [13, 14]. Inconsistent results are however reported [15, 16]. Salles et al. [17] state that strength training directly affects the functional capacity of dynamic stabilizers, which results in increased joint stability and consequently, reduced injury rates. They then concluded that strength training at the same intensity improves JPS compared to different intensity training, which improves muscle spindle sensitivity and hence improves neuromuscular control in the shoulder; furthermore they demonstrated that closed kinetic chain (CKC) exercises increase and/or restore the dynamic stability of the shoulder by facilitating the co-activation of the shoulder muscles caused by the joint approximation [18, 19]. Open kinetic chain (OKC) exercises but increase the proprioception by emphasizing awareness of the joint position [20]. Padua et al. [21] however, stated that CKC exercise, OKC exercise and Proprioceptive Neuromuscular Facilitation (PNF) are not able to improve shoulder proprioception and neuromuscular control in young men and women.

FIFA has already developed a shoulder prevention programme called the “FIFA 11 + shoulder” (11 + S) programme for goalkeepers [22]. The programme consists of three sections: general warm-up, exercises to improve the strength and balance of shoulder muscles, elbows, wrists and fingers; and advanced exercises for core stability and muscle control. The 11 + S programme was initially intended for soccer goalkeepers, the programme however could be recommended for players of other overhead sports as well [22, 23], and since volleyball is also one of the overhead sports, although with different movement pattern comparing to goalkeepers but still volleyball players have some similarity using their shoulder over their head [24, 25], and also the defect of shoulder proprioception and dynamic stability in volleyball players increase the need of the sensory-motor system for neuromuscular control and the feed forward and feedback mechanisms are considered as critical points of the kinetic chain, making their training extremely important for the prevention of injuries, we therefore hypothesize that the 11 + shoulder program can also have beneficial effect on the proprioception and stability of the volleyball players’ shoulder. Considering the importance of proprioception for joint stability and injury prevention, the purpose of this study was to investigate the effect of eight weeks of the 11 + S injury prevention programme on shoulder proprioception and stability in Iranian young male volleyball players.

## Methods

### Design

This study was a Pre-test – post-test quasi-experimental cohort design with a control group aiming to investigate the effect of an eight-week 11 + S programme on shoulder proprioception and stability in young male volleyball players. Two teams were randomly divided into intervention and/or control group.

### Participants

Two teams consist of Thirty-two young male volleyball players (mean age 17.5 ± 1.47 years) Were selected by available methods from thirteen teams of the Iranian Youth Volleyball Premier League volunteered to participate in this study during the pre-season. Block randomization method was used to create a random sequence. In this method, 4 blocks of all possible combinations (6 possible modes AABB, ABAB, ABBA, BBAA, BABA, BAAB) were created. Then these blocks were numbered and randomly selected and placed one after the other, thus, the participants were divided into two groups A (intervention = 16) and B (control = 16). MZ generated all the steps of random allocation sequence, enroll and assignment of participants to the interventions. The sample size was estimated based on the findings of previous studies [15, 26–28]; so that using the G-Power software with 95 % power at the 0.05 level of significance the expected number of participants was estimated (Considering a full factorial ANOVA and using small effect size (h = 0.53), confidence level (a = 0.05), and desired power (95 %) for 2 measurements, a test power analysis was performed and the required total sample size was calculated to be 32 subjects).

In such studies, blinding cannot be performed completely and therefore, in the present study, a single-blind method was used where only participants were tried to be blinded from the study. For this purpose, both intervention and control groups were given warm-up exercises. The warm-up exercises of the intervention group were the main exercises of 11 + S, while the warm-up exercises of the control group were the same as their normal warm-up exercises.

Inclusion criteria for the study were as following: (1) Having no severe injuries (more than three weeks absence from exercise) over the past six months, (2) Having at least three years playing experience in volleyball, (3) Exercising approximately three sessions per week including matches and training. The absence in two consecutive training sessions, and conducting any systematic injury prevention programme leaded to players’ exclusion from the investigation.

### Ethical considerations

This study was approved by research ethics committee of sport science research institute of Iran. Written consent was obtained from the participants before participation at the study. Participants had the right to withdraw from the study at any time without any consequences.

### Procedures

#### Demographic data

All the participants filled into the questionnaires on the age, height and weight, previous shoulder injuries, their specific game post, game level and training hours.

#### Proprioception measurement

The Biodex System 4 dynamometer (Biodex Medical Systems, New York, USA) was used to measure the proprioception of the dominant shoulder. Two methods of TTDPM and JPS in passive mode were used to measure proprioception. We used the passive protocol for both because the proprioception measurement is reported to have a greater reliability )ICC values ± SD: 0.92 ± 0.07 (in the passive protocol compared to active protocol (ICC values ± SD: 0.34 ± 0) [10]. The dominant side was determined using the Edinburgh questionnaire [29, 30]. The subjects did not perform any exercise the day before the test. Before each testing session, the dynamometer was set in accordance with the manufacturer’s recommendations. A standardized testing protocol was followed. Tests were conducted in the sitting position. To provide stability and prevent extra movements, participants were fixed with straps around the shoulders, chest, and hip. To remove visual feedback and auditory feedback the blindfold and headphones were used during the test and subjects listened to white noise during the test. In order to familiarize the participants with the levels, the test was performed twice before the start. All tests were performed between 9 am and 2 pm. For each individual, pre and post tests were taken approximately at the same time of the day with the same manner and order.

To determine the JPS, shoulder internal/external rotation were measured at 2 degrees per second in passive mode. The rationale for choosing these velocities was based on previous studies in which comparable velocities were used [11].

**Fig. 1:**
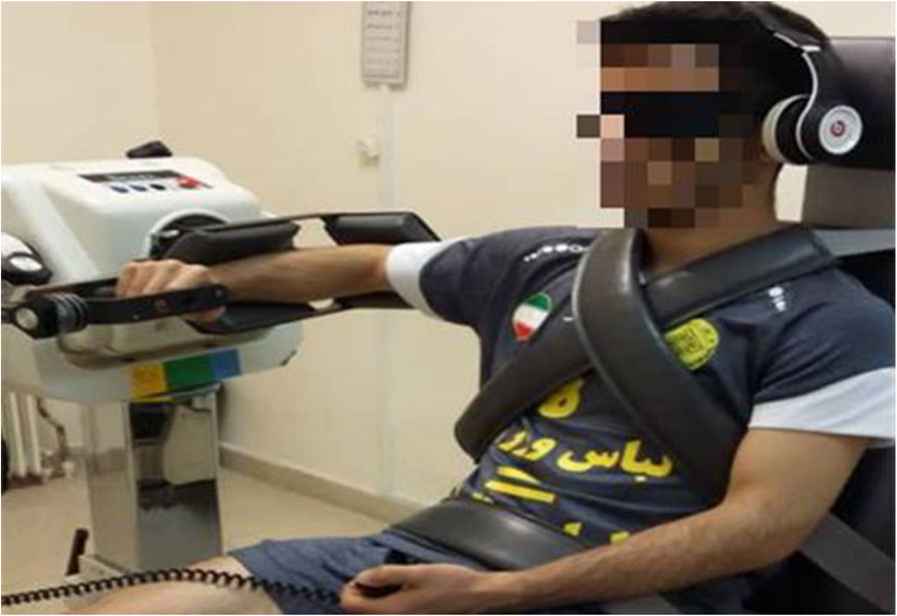
Setup for assessing proprioception of shoulder external and internal rotation using the isokinetic dynamometer

The dominant shoulder was positioned at 90 degrees of abduction and 0 degrees of external rotation (ER) in the scapula plane (30 degrees ahead of the frontal plane), as the measurement of the proprioception of internal and external rotators at this angle has high validity [10]. The elbow flexed 90 degrees. The forearm was in internal rotation (IR); see Fig. 1. The target angle was 45 degrees of IR (from neutral to 45 degrees of IR) and 75 degrees of ER (from neutral to 75 degrees of ER). The limb moved up to 45 degrees of IR by the device and the shoulder was kept in this position for 10 s and the participant was then asked to focus on the position. Then the manual key was given to the participant and the device information on the motion was inactivated by 45 degrees of IR and 75 degrees of ER, and the participant was asked to press the key at any angle he felt that he reached to the target angle. Three repetitions for IR and three repetitions for ER were performed and the magnitude of the difference between the reconstructed angles and the target angle was calculated and considered as the angle reconstruction error [11].

To determine the TTDPM, shoulder internal/external rotators muscles were measured at 0.25 degrees per second in passive mode. The dominant shoulder position was similar to the JPS test (Fig. 1). Then the manual key was given to the participant and the shoulder passively started from 0 degree to the IR direction and the person was asked to press the key as soon as the movement was detected. Then the onset test and the mean motion detection threshold were recorded 3 times in the test [31]. The same protocol was then repeated for ER.

#### Dynamic stability measurement

The Upper Quarter Y Balance Test (UQYBT) was used to measure the dynamic stability of the dominant shoulder. The UQYBT is a valid and reliable test (with ICC coefficients ranging from 0.80 to 1.0 for test-retest as well as intra-rater reliability) [32, 33] for measuring unilateral (dominant hand) upper extremity performance and stability in a closed-chain position. It can identify upper extremity motor limitations and asymmetry and therefore can be used to predict injury in athletes [33].

**Fig. 2:**
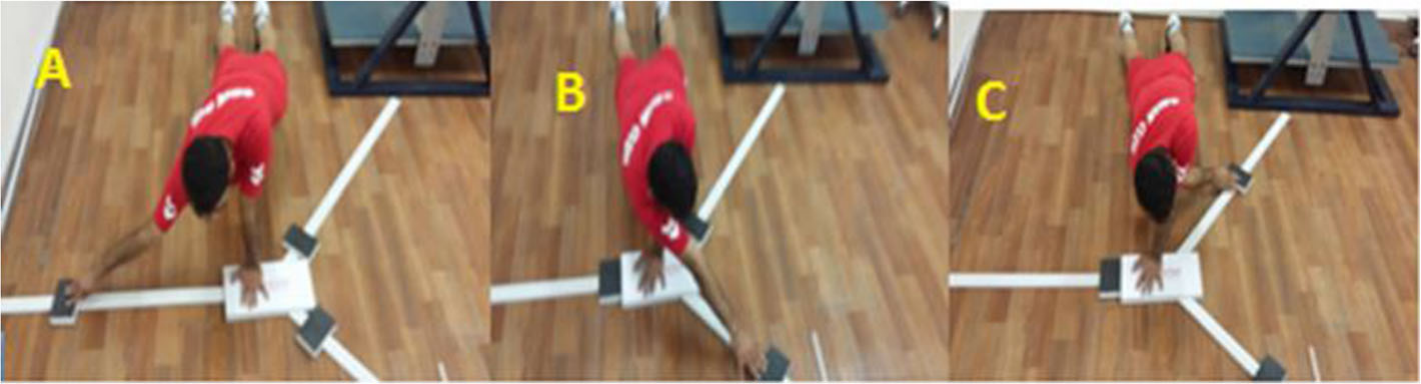
Upper Quarter Y-Balance Test - Direction of reach is named relative to the stationary upper extremity (**A**. Medial Reach Direction, **B**. Superior Lateral Reach Direction, and **C**. Inferior Lateral Reach Direction)

To perform this test, the participant was asked to place the thumbs on the palms of the fingers and elbows open, keeping the spine and lower limbs in one position. The location of the thumb was indicated by a line and the legs were about the shoulder-width apart (the legs were not more than 30 cm apart). In this situation, the participant was asked to maintain the position of the support arm, trunk, and lower limb, to reach the medial, supero-lateral and infero-lateral directions[34] as far as possible with his free hand (Fig. 2). In order to be able to compare the results of this study with others, The player’s upper limb length reach values (the seventh cervical vertebra to the end of the longest finger at 90 degrees shoulder abduction and extension of the elbow, wrist, and toe) were normalized [35]. While maintaining the push up position the ability to reach all three directions was measured without rest and without touching the ground. The participant was allowed, after each round of reaching all 3 directions, to place the free hand on the ground and rest [33]. Before the test, each participant was allowed to perform two practice trials. Three consecutive trials in all three directions were performed on the dominant arm. In each direction, the highest reach was recorded and was calculated in the following formula to calculate the overall composite score [35]:

$$\mathrm{Combined}\;\mathrm{score}\;=\;(\mathrm{middle}\;\mathrm{access}\:+\:\mathrm{lower}-\mathrm{external}\;\mathrm{access}\:+\:\mathrm{upper}-\mathrm{external}\;\mathrm{access})\;\div\;(\mathrm{upper}\;\mathrm{limb}\;\mathrm{length}\;\times3)$$

### Intervention programme

The 11 + S programme was developed by an international group of experts, including orthopedics, physiotherapists, and sports rehabilitation specialists. The programme focuses on core stability, neuromuscular control, eccentric rotators’ strength, and shoulder agility. It consists of three parts: general warm-up (part I); strength and balance training for the shoulders, elbows, wrists, and fingers (part II); core stability and muscle control exercises (part III). The second part of the programme has three levels of difficulty and to achieve that elastic bands at three resistance levels (blue [low], black [medium], and gold [high]) are used. Prior to the implementation of the 11 + S programme by the participants of intervention group, several educational sessions were organized for the coaches by the researcher to familiarize the coaches with the exercises and how they are implemented, this was done in order to ensure that the programme would be properly implemented by the coaches, Informative posters were also provided. The researcher supervised the training at intervention group at each session to validate the implementation of the 11 + S injury prevention programme. The content of the training and participants’ progress was monitored by the researcher every two weeks during the intervention period.

All players started training from level one and moved on to the next level if they were able to do error-free training determined by coaches under standard conditions. The participants at the intervention group performed 11 + S exercises three times per week as their warm-up protocol. The 11 + S programme was usually taken about 20–25 min.

The control group also performed their normal warm-up for 25 min, including 5 min of stretching exercises for the whole body, then dynamic warm-up exercises for 10 min, including running and jumping movements, and finally 10 min of exercises with the ball, including spiking, etc.

In order to avoid the effect of fatigue on the proprioception due to increased intramuscular concentrations of lactic acid, bradykinin, arachidonic acid, and serotonin after fatiguing contractions which may affect the muscle spindle system, and, thus, proprioceptive acuity [28], players performed JPS and TDDPM tests with an isokinetic dynamometer before the UQYBT test. Before the pre-test and post-test, the players performed a standardized 5-minute warm-up on an arm-cycle ergometer (Monarch Model 894E, Sweden) at a self-determined cadence (between 80 and 110 rpm) with the workload set to 75 W. Participants then familiarized with the dominant shoulder test to learn how to perform a proprioception test with an isokinetic device. They tried to do the UQYBT test, did the test twice in three directions. The proprioception and UQYBT tests for the dominant shoulder were conducted in standard conditions. All tests were conducted three days before and three days after the intervention programme at the Shahid Beheshti University Sport Laboratory.

### Statistical analysis

All statistical analyses were performed by SPSS 24.0 software (IBM corp. Amork, NY). Descriptive data are provided as mean and standard deviation. The demographic characteristics of the participants of two groups at baseline were analyzed using the independent samples T-test. Measurements of proprioception in internal and external shoulder movement (for JPS and TTDPM) have been reported. Two-factor ANOVA test (condition factor: “pre” and “post” and group factor: intervention vs. control) with a group x condition interaction was used to analyze the within and between group evaluation over the eight-week intervention period at 95 % significance level with alpha equal or less than 0.05. To limit the possibility of getting a statistically significant result, Bonferroni adjustment for multiple comparisons was used for post hoc test. To analyze the effect of the intervention on the different proprioception measures, we calculated the mean differences and the ∆%between the intervention group and the control group. The effect size was calculated using Cohen’s d value. An effect size between 0.2 and 0.5 was considered a small effect, between 0.5 and 0.8 a medium effect, and greater than 0.8 a large effect [36].

## Results

**Fig. 3:**
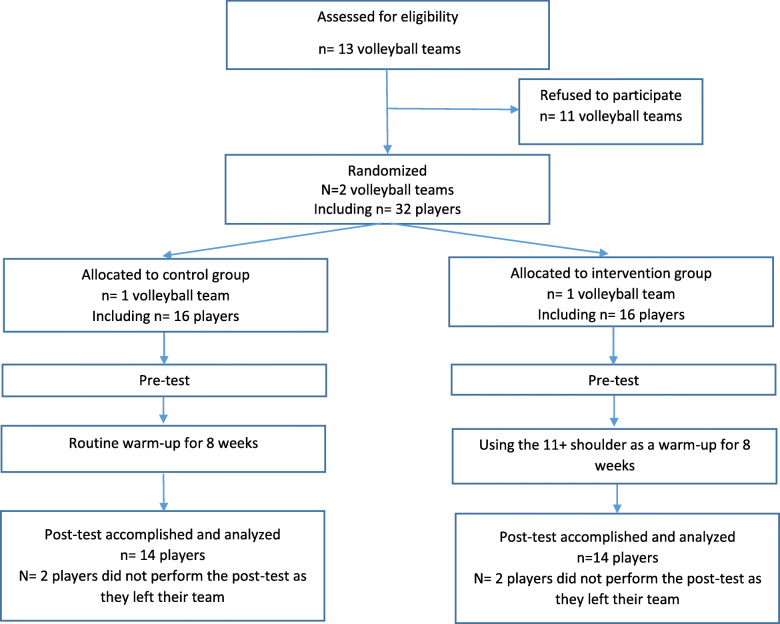
Flow of the study

Four out of the 32 young male volleyball players, two out of each group, dropped out of the study because of leaving their teams and not attending the training sessions (see the flow of participants, Fig. 3). The data related to 28 players were analyzed (intervention group (INT), *n* = 14 and the control group (CON), *n* = 14). The participants did not suffer any physical complaints prior to the tests. No injuries induced-time-loss occurred during the period of conducting the study in any of the two groups. All players reached level three in the intervention group. The demographic characteristics e.g. age, body height and weight, Body Mass Index and volleyball experience of the two groups were not significantly different (*p* > .05) (Table 1). The participant’ maturity was determined via Tanner scales while all of the kept positions four (IV) and five (V).

**Table 1:** Mean ± SD values for player’s anthropometrics

Group	Players(n)	Age (yrs)	Height (cm)	Weight (kg)	BMI (kg.m-2)	Experience (yrs)
**Intervention**	14	17.9 (2.21)	1.82 (0.01)	71.58 (10.64)	21.39 (1.65)	4.07 (1.5)
**Control**	14	17.09 (0.73)	1.86 (0.01)	75.06 (12.36)	21.52 (2.13)	4.35 (1.7)

Between subject repeated measures ANOVA showed no significant interaction effect between the time × groups in none of the proprioception (JPS and TTDPM) variables (Table 2). However, there was a main effect of time in the JPS of the shoulder IR motion at -45 degree (F_1, 26_= 21.31, *p* < 0.05) and the JPS of the shoulder ER motion at + 75 degree (F_1, 26_= 23.11, *p* < 0.05) and the TTDPM of the shoulder ER motion at + 75 degree (F_1, 26_= 5.35, *p* < 0.05).

**Table 2:** Mean values (SD) of Passive JPS, TTDPM test and UQYBT for 11 + S intervention (INT) and the control group (CON) with 95 %-CI

	INT (*n* = 14)		CON (*n* = 14)		F	time*group interaction p value	ηp2_time*group_	Time *p* value	Group *p* value	ηp2_time_	ηp2_group_
	**Pre**	**Post**	**Pre**	**Post**							
**JPS 45° IR**	9.21(3.99)	5.42(3.01)	8.64(4.33)	5.57(4.57)	0.23	0.63	0.009	0.001	0.87	0.45	0.001
**JPS 75° ER**	11.42(4.77)	5.36(2.55)	10.76(4.41)	7.57(5.30)	2.23	0.14	0.07	0.001	0.57	0.471	0.013
**TTDPM IR**	2.94(1.70)	1.69(0.98)	2.1(0.58)	1.98(0.83)	3.71	0.06	0.12	0.02	0.36	0.171	0.032
**TTDPM ER**	1.75(1.07)	1.55(1.19)	1.55(1.17)	1.12(0.51)	0.39	0.53	0.01	0.09	0.38	0.104	0.03
**UQYBT**	0.77(0.04)	0.85(0.06)	0.76(0.07)	0.81(0.08)	5.11	0.03*	0.16	0.001	0.403	0.685	0.027

Legend: *INT *Intervention Group, *CON *Control Group, *JPS *Joint Position Sense, *TTDPM *Threshold to detect passive motion, *UQYBT *Upper Quarter Y Balance Test, *IR *Internal Rotation, *ER *External Rotation

*indicates significant effect (*p* < 0.05)

There was a statistically significant interaction effect of time × group on the dynamic stability (F_1, 26_= 5.11, *P* = 0.03, ηp^2^ = 0.16). Dynamic stability was found to improve from pre- to post-test in the intervention group (0.77 ± 0.04, 0.85 ± 0.06 respectively).

## Discussion

Although 11 + S was useful for soccer goalkeepers, and given that this program is also recommended for players in other overhead sports, our hypothesis was that the 11 + shoulder program has an effect on shoulder proprioception and shoulder stability of volleyball players as well.

The most important finding of this study was that following an eight-week 11 + S warm-up programme, the dynamic stability of shoulder increased in young male volleyball athletes. Based on the findings of this study our hypothesis was therefore confirmed.

Since this is the first study to investigate the effects of the 11 + S programme on the proprioception and dynamic stability of shoulder in young male volleyball players, no other similar study is available to compare the results with.

Regarding to the findings related to Y-Balance test in line with our study, Amirkolahi et al. (2019) in the study of the effect of Swiss ball training on the integration of functional movements and balance of adolescent badminton players demonstrated that eight weeks Swiss ball training increases the FMS and Y scores of the lower and upper limbs. They therefore considered the increase in the reach scores after this training as a key role in preventing injury among badminton players. In their study, exercises such as plank, press-up, etc. were used, which are very similar to several drills of 11 + shoulder exercises in terms of function and muscle involvement, which can be considered as a reason to improve the Y-balance scores in the present study.

The neuromuscular exercises improves the ability of the nervous system to produce a fast and desirable muscle stimulus pattern that enhances dynamic joint stability and the reduction in forces on the joint and the release of movement patterns [37]. The significant increase in stability of the participants on this study can be attributed to the effect of the neuromuscular and plyometric exercises involved in the 11 + S programme. On the other hand, according to the researchers, plyometric exercises increase the excitability of the nervous system and the reactive capacity of the healthy athletes shoulder neuromuscular system [22].

The findings of our study further indicated that an eight-week 11 + S warm-up programme would have no effect on shoulder proprioception (JPS and TTDM) of young male volleyball athletes. These results are consistent with those reported by Lin et al. [15] who examined the effect of the rotator cuff and scapula strength training on the joint position sense in healthy subjects. Lin et al. [15] demonstrated that these exercises had no effect on shoulder JPS in the intervention group. They have however stated that strength training may have an impact on the shoulder JPS in people with shoulder injuries. Furthermore, Dilek et al. [38] and Naughton et al. [39] have also demonstrated that exercise training can improve shoulder JPS in patients suffering from shoulder impingement syndrome and patients with posterior shoulder displacement. In contrast, Fortun et al. [40] reported that running eight weeks of Plyometric training programme on healthy but overhead athletes had no significant effect on proprioception of shoulder internal and ER motion. It can be hypothesized that, chronic pain and inflammation may have affected e.g. deteriorated the central and peripheral JPS [41], and conducting exercise training on the other hand then might have recovered JPS in patients with shoulder injury by reducing pain and symptoms related to their situation [42]. It can therefore be suggested that although exercise training for people with shoulder injuries may improve their JPS, however such effect is not expected in healthy people and it will not have much effect on the JPS.

Another interpretation attributed to the controversy between previous studies investigating the effect of exercise training on proprioception would be the difference between the type and mode of the exercise training and the test applied in these studies. Conducting active testing techniques for assessing proprioception can lead to different results; Swanik et al. [16] who used an active technique to assess shoulder proprioception concluded that plyometric exercise training increases the proprioception of the shoulder joint. Salles et al. [17] who applied 4 types of strength training including chest press, lat pull, shoulder press, and rowing and assessed shoulder JPS via the Joint-Position Reproduction Test reported similar results. Finally, the 11 + S training protocol includes OKC and CKC exercises (e.g. push-up and walking on the hands), the efficiency of these exercises on proprioception is reported inconsistent in different studies. Salles et al. [17] and Rogol et al. [43] reported that these exercises are effective for improving the shoulder proprioception. However, Padua et al. [21] who examined the effect of five weeks of CKC and OKC exercises on shoulder JPS found that these exercises did not have any significant effect on proprioception and neuromuscular control of the shoulder; they therefore suggested that CKC exercises should not be considered as an effective approach to facilitate proprioception and neuromuscular control.

## Conclusions

Results of this study suggested that 11 + S injury prevention programme can improve the dynamic stability of the volleyball players’ shoulders. However, there was no evidence indicating the positive effects of 11 + S injury prevention programme compared to a regular warm-up in improving the proprioception (JPS and TTDPM) of shoulder.

## Data Availability

The data that support the findings of this study are available on request from the corresponding author.

## Abbreviations

JPS: Joint position sense
TTDPM: Threshold to detect passive motion
CKC: Closed kinetic chain
OKC: Open kinetic chain
11 + S: FIFA 11 + shoulder programme
UQYBT: Upper Quarter Y Balance Test
INT: Intervention group
CON: Control group
IR: Internal Rotation
ER: External Rotation
